# Navigating sociocultural practices and traditions in HIV management: a review of African cultural barriers to achieving sustainable development goal target 3.3

**DOI:** 10.3389/fepid.2026.1710531

**Published:** 2026-03-05

**Authors:** Reneilwe G. Mashaba, Cairo B. Ntimana

**Affiliations:** 1 DIMAMO Population Health Research Centre, University of Limpopo, Polokwane, South Africa; 2 Department of Pathology, University of Limpopo, Polokwane, South Africa

**Keywords:** health, HIV, SDG, sociocultural barriers, traditional medicine

## Abstract

The narrative review aimed to explore how the sociocultural belief systems influence the health-seeking behavior of individuals living with HIV (late ART initiation and treatment discontinuation) and the subsequent impact on SDG Target 3.3. We searched PubMed, using a search strategy using keywords such as “HIV management barriers,” “SDG Target 3.3,” and “sociocultural beliefs”, and it was adapted on Google Scholar, and AJOL between 1st may to 30th June 2025. Findings demonstrate that pluralistic health-seeking behavior, such as sequential use of biomedical care, religious healing, and traditional medicine, persists amongst individuals living with HIV. This is informed by society, religious, and traditional healers. The pluralistic health-seeking behavior is practiced based on what the individual perceives as the causes of HIV, the influence of religion and faith leaders, and traditional claims of HIV cure. Although pluralistic health-seeking behavior may offer emotional support, they associated with delayed initiation, disruptions, and adherence to ART, inadequate retention in care, and lower likelihood of long-term viral suppression, weakening the HIV care continuum. Although emerging research has explored the potential role of traditional medicine in HIV management, there is a lack of evidence to support its use as a standalone treatment. The findings of this review, emphasizes a need for a structured collaborative care models. Formal engagement and dialogue amongst traditional, religious leaders, and PHC practitioners’, development of referral linkages and integration of culturally sensitive HIV education within existing health systems at a policy level should be explored.

## Introduction

The Sustainable Development Goal (SDG) Target 3.3 outlines a global commitment to end the HIV epidemic by 2030 (1, 2). However, progress towards achieving this goal is hindered by sociocultural factors in certain settings, including Sub-Saharan Africa (SSA). These sociocultural factors and practices have been reported in the literature to influence health-seeking behaviors in diverse contexts (3). From a social determinant of health perspective, such factors shape individuals' access to care, decision-making processes, and engagement with biomedical health services (4–6). Although biomedical advancements have significantly improved HIV outcomes, their effectiveness is mediated by the social and cultural environments within which they are implemented across different communities (7). According to the UNAIDS, SSA accounts for about two-thirds of the global HIV burden (8). Although biomedical interventions over the years have reduced HIV from a death sentence to a manageable condition, their success is often hindered by sociocultural factors, such as stigma, traditional healing practices, and religious beliefs (9). These factors can be understood within health-seeking behaviour models, which highlight how perceived beliefs, social norms, and trust in health systems influence care-seeking choices in specific sociocultural settings (3, 10). Together, these dynamics illustrate how sociocultural barriers intersect with biomedical progress rather than operate independently. These cultural barriers contribute to undermining progress toward SDG 3.3 (11).

In many SSA contexts, HIV-related stigma, which discourages People living with HIV (PLHIV) from seeking medical attention in the form of testing, initiating treatment, taking treatment consistently, or disclosing their status to their loved ones for support, for fear of social exclusion (8). Within the framework of stigma theory, such experiences reinforce secrecy, delayed care, and poor treatment adherence in affected populations (12). Beyond stigma, other sociocultural influences further shape how individuals navigate HIV care pathways (13, 14). For instance, SSA has an increased number of faith healers and traditional healers who are held in high regard by the community members (15). As a result, the teachings of these individuals are sometimes upheld without question within those settings (15). This coexistence of biomedical care with traditional and religious healing systems reflects medical pluralism, where individuals navigate multiple health systems simultaneously, depending on cultural norms and personal beliefs (16, 17). These teachings (traditional and religious) sometimes go against the biomedical recommendations, with some communities prioritizing herbal medications and spiritual healing over antiretroviral therapy (15). Furthermore, in certain religious contexts, teachings discourage condom use, thus ultimately promoting risky sexual behaviors (18). Collectively, these interconnected beliefs and practices contribute to inconsistencies in HIV prevention and treatment engagement in specific sociocultural environments (19). Such dynamics help explain the persistent gap between the availability of HIV interventions and their real-world effectiveness (19). Conceptualizing these barriers through established frameworks allows for a deeper understanding of how sociocultural contexts shape HIV management outcomes (20, 21). It is important to understand cultural barriers to design inclusive, context-sensitive strategies that align with Africa's diverse sociocultural landscape (22). While previous studies have extensively examined HIV-related stigma and the role of traditional and religious healing practices, much of this literature remains fragmented, context-specific, or focused on single sociocultural dimensions (23, 24). There is limited synthesis that integrates these factors across African regions while explicitly situating them within global development targets such as SDG 3.3. This narrative review aimed to explore how traditional practices and beliefs impact HIV management across African regions, and their implications for achieving SDG 3.3. The findings of this review are expected to contribute to a policy-relevant perspective on monitoring progress toward the 2030 agenda.

## Review methods

This study adopted a narrative review design to synthesize existing evidence on sociocultural practices, beliefs, and traditions influencing HIV management in Africa, and their implications for achieving Sustainable Development Goal (SDG) Target 3.3. In order to facilitate the conceptual integration of heterogeneous evidence qualitative, quantitative, mixed-methods, and policy-oriented literature, that might not be amenable to systematic review or meta-analysis, a narrative approach was chosen.

### Search strategy and period

A comprehensive literature search was conducted between 1 May and 30 June 2025, covering studies published from database inception to June 2025. Databases searched included PubMed, Google Scholar, and African Journals Online (AJOL) to ensure coverage of both international and Africa-focused peer-reviewed literature.

Search terms were combined using Boolean operators and included: “HIV management,” “socio-cultural beliefs,” “traditional medicine,” “religion,” “health-seeking behaviour,” “medical pluralism,” “Africa,” and “SDG Target 3.3.” Reference lists of included articles were also reviewed to identify additional relevant studies.

### Eligibility criteria

Studies were included if they:

1. Examined sociocultural practices, beliefs, or traditions influencing HIV prevention, testing, treatment initiation, adherence, or retention in care;
2. Were conducted in Sub-Saharan African settings;
3. Employed qualitative, quantitative, mixed-methods, or policy/implementation study designs;
4. Were peer-reviewed, published in English, and available from database inception to 2025.

Studies were excluded if they were dissertations, conference abstracts, preprints, or opinion pieces without analytical grounding, or if they did not explicitly address sociocultural dimensions of HIV management.

### Study selection and included evidence

Titles and abstracts were screened for relevance, followed by a full-text review of eligible articles. In total, 17 studies met the inclusion criteria and were included in the final narrative synthesis. The included literature comprised qualitative studies and cross-sectional studies relevant to HIV management in Sub-Saharan Africa (Supplementary Table 1).

### Data extraction and thematic synthesis

Key sociocultural constructs and study characteristics (country, population, and study design) were the main focus of the manual data extraction process. Themes emerged from the data thanks to the use of a primarily inductive thematic analysis approach. Deductive framing based on accepted theories of stigma, medical pluralism, and health-seeking behavior was used to supplement this process. To evaluate their implications for the HIV care continuum and SDG Target 3.3, identified themes such as pluralistic health-seeking behavior, perceived causes of HIV, traditional and faith-based healing practices, stigma, and cultural silence around sexuality were narratively synthesized.

### Quality appraisal

Formal quality appraisal was not undertaken, as the primary aim of this narrative review was conceptual and contextual synthesis rather than evaluation of intervention effectiveness. Consistent with narrative review methodology, studies were included based on relevance, contextual depth, and contribution to understanding sociocultural influences on HIV management across African settings.

## Review findings

### Pluralistic health-seeking behavior

Evidence from several studies in African communities reported that approximately 70% of patients, and individuals often combine traditional and biomedical healthcare services (25, 26). This plurality in health-seeking behavior is informed by what participants perceive as the cause of their condition (27). When symptoms persist individuals often begin with home-based remedies, including over-the-counter medicines and herbal preparations (28). When the symptoms persist, care may be sought from traditional healers (THs), health facilities (HFs), or both, including in the context of HIV-related illness (28). Traditional healers frequently remain the first pint of contact for many individuals, to familiarity, trust, accessibility, and beliefs surrounding the nature, cause, and severity of illness (29). For example, when HIV is perceived to be caused by witchcraft or supernatural forces, individuals may preferentially consult THs, who are believed to have the capacity to address such causes (30).

Because THs are not authorised to provide HIV testing, individuals may remain unaware of their HIV status while pursuing alternative treatments (31). Movement through home remedies, traditional healing, and biomedical care forms a health-seeking pattern that has been reported to contribute to delays in HIV diagnosis, increasing the likelihood of detection at a more advanced disease stage (31). Delayed diagnosis has been associated with later initiation of ART (31). Individuals who attribute HIV to supernatural causes are less likely to initiate or adhere to ART, particularly when biomedical treatment is perceived as ineffective against spiritual illness (32).

**Figure 1:**
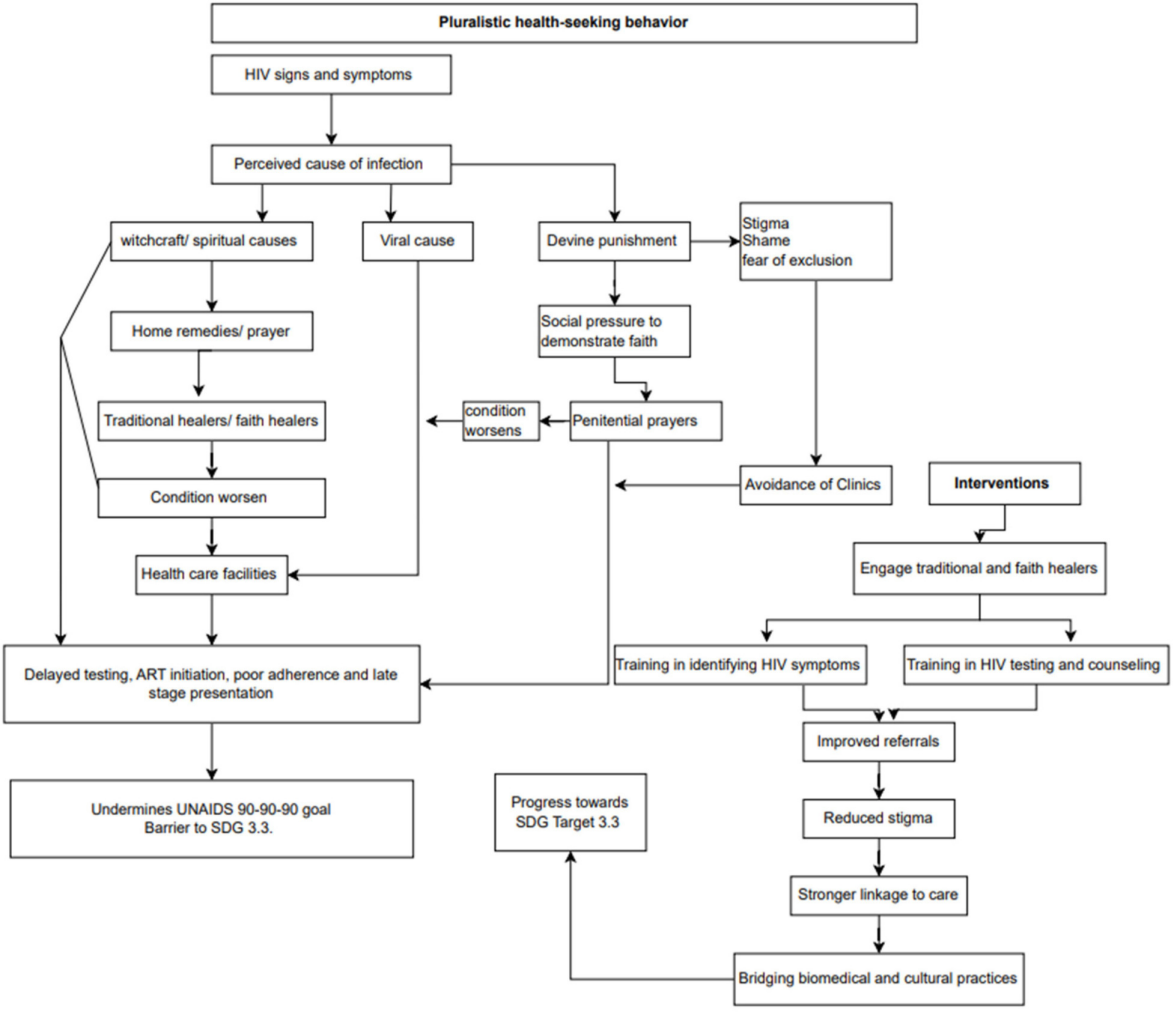
Health-seeking behavior and interventions.

Although HIV testing services are widely available in health facilities, late HIV testing has been repeatedly reported as a barrier to effective HIV management (31). Delayed testing has been associated with delayed treatment initiation and reduced likelihood of sustained viral suppression (31, 33). In addition, lack of awareness of HIV status has been associated with lower condom use and engagement in high-risk sexual behaviours (34, 35). Late HIV diagnosis has been further associated with increased morbidity, mortality, and healthcare costs (31). Therefore, to end the epidemic of AIDS, tuberculosis, malaria, and neglected tropical diseases by 2030 (Sustainable Development Goal Target 3.3), health-seeking behavior specific to the communities should be considered when engaging the community for better outcomes of HIV management (36). Effective HIV responses in African contexts, therefore, require a balanced approach that recognises both the challenges and the strengths of traditional and religious systems, prioritising collaboration, capacity-building, and referral over exclusion or confrontation. Collectively, the reviewed studies indicated that pluralistic health-seeking behaviour is consistently associated with delayed HIV testing and delayed initiation of ART, which in turn reduces the likelihood of sustained viral suppression and undermines progress toward achieving SDG Target 3.3 in African settings (Figure 1).

### Perceived causes of HIV, traditional claims of HIV cure, advancements in conventional medicine research, and collaborative care

Some individuals believe that HIV is a disease that has evolved from older, mystical diseases which only THs can cure, while some claim that HIV is witchcraft-induced or a mysterious disease with HIV-like symptoms (25). Individuals holding such beliefs may spend extended periods seeking traditional or spiritual remedies, thereby delaying access to ART (37). Consequently, individuals may delay HIV testing or initiating treatment, which increases the risk of advanced disease progression, ongoing transmission, and undermines the first and second “90s” of the UNAIDS 90-90-90 targets (early diagnosis and sustained treatment) (31). Some individuals discontinue attendance at health facilities, believing biomedical care cannot cure HIV, while others use traditional medicine concurrently with ART or discontinue ART altogether in favour of herbal treatments (38). The potential drug interaction between herbal medication and ART remains to be studied in these settings. Literature shows that people who believe HIV can spread through witchcraft and other supernatural means are less likely to practice safe sex, including the use of a condom (30).

While these traditional beliefs can hinder access to biomedical care, they have also prompted scientific interest in certain medicinal plants used in African traditional healing systems (39). Research has explored the antiviral properties of plants such as Sutherlandia frutescens, Hypoxis hemerocallidea, and Moringa oleifera, including their potential effects on reverse transcriptase or protease enzymes, immune modulation, and HIV-related wasting (39, 40). Although these plants have yielded positive results, current evidence is insufficient to support their use as standalone treatments for HIV (41). Furthermore, extensive research is still needed to validate their properties. Reliance on these remedies in place of antiretroviral therapy could jeopardize health outcomes and delay progress toward ending the HIV epidemic by 2030 (39).

As already mentioned above, most Africans consult both THs and HFs. This dual health-seeking behavior reflects not only accessibility and cultural preferences, but also the belief that spiritual and biomedical causes of illness must be addressed simultaneously (42). Recognizing this cultural complexity, there have been growing calls for integrative strategies that bridge traditional and biomedical healthcare models (43). What then has been done to navigate/collaborate with African cultural representatives/stakeholders to bypass these barriers to achieving Sustainable Development Goal Target 3.3?. The World Health Organization (WHO) has acknowledged the impact of traditional medicine and encouraged member states, particularly in Africa, to integrate traditional health practitioners (THPs) into primary health care systems (39). This is highlighted in the Traditional Medicine Strategy (2014–2023) that supports regulatory frameworks, training, and collaboration between biomedical and traditional systems (44–46). Such frameworks aim to respect traditional practices while ensuring that patients receive timely, evidence-based, essential care for improving diagnosis rates, linkage to care, and treatment adherence (47). Some countries within Africa have started/ created frameworks to integrate traditional healers into primary health care systems for better management of HIV and other morbidities (39).

For instance, in South Africa, the Traditional Health Practitioners Act was passed (48). This act aims to establish a Traditional Health Practitioners Council of South Africa, provide a regulatory framework for traditional health care services, and manage the registration, training, and conduct of practitioners in this field (39). Although this legislation is a promising step toward formal integration, its full implementation remains pending due to delays in establishing the regulatory council (39). This delay highlights the gap between policy intention and practical execution, which continues to limit the potential for collaboration between traditional and biomedical practitioners (49). Even though that is the case, some progress has been made in academia and research spaces in South African universities. For example, the Wits University's MRC/Wits Agincourt Research Unit conducted a study that aimed to reduce the incidence of HIV by empowering traditional healers to conduct HIV testing and refer those positive to the primary health care facilities for treatment (50). Also, to foster a collaboration amongst traditional healers, community healthcare workers, and the Department of Health to strengthen the referral system (51). Likewise, the University of KwaZulu-Natal has conducted training workshops with THs on HIV/AIDS, TB, and mental health. This was informed by the patients' right to choose where they want to consult (traditional medicine or clinics) (52). The training included skills to identify diseases and counsel their patients (53). Uganda has also conducted such trainings and reported an increase in patient referrals to hospitals for treatment and improved community trust in health services (54).

Similar efforts in Uganda, Tanzania, Ghana, and Zambia show that training traditional healers to recognize symptoms, encourage testing, and make timely referrals can lead to improved health-seeking behaviors and reduced stigma within communities (55–57). Despite ongoing cultural and systemic challenges, these collaborative models represent a critical opportunity to align culturally resonant care with global HIV goals. Strengthening these partnerships is essential to accelerate progress toward Sustainable Development Goal 3.3 and ensure no one is left behind (58). While beliefs in supernatural causation may delay engagement with biomedical care, several studies also report examples where traditional practitioners have supported HIV testing, reinforced the importance of continued ART use, and collaborated with health facilities when engaged through structured and respectful partnerships (Figure 1).

### Influence of religion and faith leaders

A similar trend of attributing HIV infection to spiritual forces has also been observed in other religious cycles and faith leaders (42). This phenomenon is not unique to one community; similar beliefs have been reported across different religious circles in Africa (30). Some religious communities' belief that those who are HIV-infected have not followed the word of God and that HIV is a punishment from God for sinful behavior or caused by “instigations” of Satan (42). The performance of rituals to exorcise malevolent spirits “disguised as a virus” are commonly practiced with the aim of healing this disease (32). At the community level, no clear-cut boundaries exist between the different systems of beliefs (traditional, spiritual, or western medicine) relating to HIV management and such boundaries are easily crossed (42). In some churches, for instance, the use of ART is discouraged because of the belief that God is the creator and the healer (30).

In certain African contexts, spiritual, traditional, and biomedical explanations for illness coexist, and individuals may move fluidly between these systems when seeking care (42). With this belief, some do not even take their sick relatives or themselves to the clinics when they are ill because faith leaders often claim HIV can be cured through prayer or spiritual healing, and/or holy rituals (30). This belief system may inadvertently discourage the use of lifesaving antiretroviral therapy, especially when religious leaders position prayer as a superior alternative to biomedical care (32). They claim, “God has the power to heal a person, but not everyone will be healed, but for those He has plans with”. This claim is different to that of THs in that it claims that God will not heal everyone but those who have faith and those God has plans for (42). Therefore, members who are infected with HIV may feel the pressure to demonstrate their faith by abandoning or not initiating antiretroviral treatment (30).

Religious leaders in some African communities are viewed as moral and spiritual authorities (42, 59). This can lead to members deferring to pastors when making personal health decisions, including those related to HIV testing, disclosure, and treatment (30). When church leaders promote faith healing and discourage the use of ARVs, patients may delay or abandon medically recommended treatments based on the recommendations of the pastor (32). A study conducted in Zimbabwe found that PLWHIV defaulted on medications because they believed in faith healing, alternative medicines, perceived spirituality as the main cause of HIV and AIDS and that they had an allegiance to church values (30). However, religious teachings do not always contradict HIV treatment; when aligned with public health messages, they can support better outcomes (42). While religious beliefs can pose barriers to ART adherence, they also offer opportunities for enhancing treatment outcomes when aligned with supportive health practices (42). However, unlike traditional healers, pastors seem to be ignored in HIV management engagements. Engaging churches and faith healing organizations should be explored (60). This is because faith leaders, like traditional healers, are deeply embedded in community life and must be seen as vital partners in HIV interventions that respect cultural contexts (30). At the same time, evidence indicates that faith leaders and religious institutions have successfully contributed to HIV prevention and care by promoting testing, reducing stigma, supporting ART adherence, and aligning spiritual support with public health guidance in collaborative programs (Figure 1).

### Stigma

HIV-related stigma is one of the hindrances in HIV management and is sometimes reinforced by religious teachings (punishment from God for sinful behavior) (61). This implies that HIV is a moral failure rather than a medical condition (62). Consequently, PLHIV may delay starting treatment or disclose to their loved ones for fear of judgment and exclusion by their community (63). In some instances, the availability of ART is seen as a driver of the epidemic, as people recover and do not show signs of the condition, they would be treated as negative. Some faith leaders are reported to have proposed stigmatizing measures (identifiable clothing) to identify, isolate those on ART, and give them drugs to reduce sexual desire (64). These indicate that the HIV related stigma remains deeply rooted in some African communities and promotes discrimination and hinders HIV management (65). Although ARVs are available in most health facilities, stigma continues to prevent some individuals from seeking treatment (66). Overall, the influence of religious belief systems remains a critical factor in shaping attitudes toward HIV, testing, disclosure, and treatment adherence. Across studies, HIV-related stigma emerges as a cross-cutting factor associated with delayed testing, poor linkage to care, suboptimal ART adherence, and challenges in achieving viral suppression, thereby constraining efforts to meet SDG Target 3.3 in African health systems (Figure 1).

### Cultural silence and secrecy around sexuality and HIV

Sex remains a taboo topic in many families and communities (67). Parents rarely talk to children about HIV, ARVs, or safe sex, particularly in Setswana and Sepedi cultures (55). This silence contributes to misinformation, poor prevention practices, and increased vulnerability because one must strike a balance between managing the conditions and hiding them. For instance, in some African cultures, communication between parents and children about sexual behavior is not common. This leads to children making mistakes because there is no one to guide them. Progressive social change takes time, and without specific efforts to educate people so that they do not misunderstand the causes of the epidemic, conservative social reactions may occur. More effort is therefore required to educate young people about healthy sexuality, openness, and safe sex. In synthesis, cultural silence surrounding sexuality and HIV is associated with limited HIV knowledge, particularly among young people, posing a sustained challenge to achieving population-level viral suppression and the targets outlined in SDG 3.3 (Figure 1).

## Discussion

This narrative review examined sociocultural practices and belief systems that influence HIV management in African contexts and assessed how these factors hinder progress toward achieving Sustainable Development Goal (SDG) Target 3.3. The findings demonstrate that pluralistic health-seeking behaviour, traditional and religious interpretations of HIV, stigma, and cultural silence around sexuality continue to shape engagement with the HIV care continuum. While biomedical interventions for HIV are widely available across Sub-Saharan Africa, sociocultural dynamics substantially mediate their uptake, effectiveness, and sustainability (3).

The review findings indicate that pluralistic health-seeking behaviour, characterised by the use of traditional healers, faith-based healing, and biomedical services either sequentially or concurrently, has significant implications for the HIV care continuum (17, 60, 68). Individuals often delay HIV testing and ART initiation while seeking alternative explanations or treatments for their symptoms, particularly when HIV is perceived to be caused by witchcraft, spiritual forces, or mystical illnesses (25, 30, 32). These delays increase the likelihood of late diagnosis, advanced disease progression, and ongoing HIV transmission (31). Furthermore, concurrent use of antiretroviral therapy and traditional or spiritual remedies, or complete discontinuation of ART in favour of alternative treatments, undermines treatment adherence and retention in care (38). This undermines the maintenance of sustained viral suppression, which is key in both personal health outcomes and the control of HIV at the population level (20). These results support the existing evidence regarding the role of sociocultural environments in the provision of HIV treatment in African settings (30, 60).

SDG Target 3.3 aims to end the HIV epidemic by 2030, a goal that is closely linked to early diagnosis, sustained ART adherence, and viral suppression (1, 2). The results of the present review indicate that sociocultural issues are an important, although sometimes neglected, barrier in the achievement of the goal in Africa. The existence of certain beliefs that view the condition of HIV infection as a spiritual/moral condition rather than a biomedical condition can lead to delays in care initiation, non-adherence, and discontinuation of care, thereby impeding the achievement of the UNAIDS treatment targets (11, 30). Stigma related to HIV infection, which is sometimes embedded in social norms and even in religious practices, still acts as a barrier in care initiation, adherence, and consistent care-seeking (12–14, 42). The silence in some cultures about sexuality and HIV infection can also be a barrier in the access of youth to accurate information about the condition, thereby increasing the risks of infection and delays in care initiation (67, 69). These issues indicate that the achievement of the goal in the region can only be possible if there is a focus on the sociocultural determinants of health (19, 22).

The review findings indicate the potential value of collaborative and integrative models of care, such as the use of traditional healers and faith leaders, and biomedical practitioners (39, 43). Although traditional medicine research has shown promise in identifying plants with antiviral and immune-modulating activity, the current evidence base does not support the use of traditional medicine as a monotherapeutic approach to the treatment of HIV (40, 41). Reliance on such therapies in place of ART may jeopardize health outcomes and delay progress toward ending the HIV epidemic (39). Importantly, traditional and religious systems should not be viewed solely as barriers to HIV care. The reviewed evidence indicates that these systems are social, adaptive, and can facilitate HIV testing, ART initiation, adherence, and retention in care, provided that collaboration, respect, and role clarity are emphasized.

However, traditional and faith leaders remain influential community gatekeepers and therefore represent critical partners in improving HIV outcomes (42, 57). Operationalising collaboration may involve structured dialogue between health systems and community-based healers, formal referral pathways from traditional and religious settings to primary health care facilities, and training initiatives that enable non-biomedical practitioners to recognise HIV symptoms and encourage testing and treatment adherence (45, 46). Evidence from South Africa, Uganda, and other African countries suggests that such collaborative approaches can improve referrals, reduce stigma, and strengthen engagement with HIV services (15, 50, 52, 56, 69).

The results of this review highlight that HIV policies and programs implemented in Africa have to transcend biomedical approaches and incorporate cultural practices. It has been suggested that policymakers have to focus on creating regulations that allow for cooperation between traditional, religious, and biomedical health systems without compromising evidence-based care for HIV patients (45). Community-based HIV education that takes sociocultural beliefs into consideration instead of dismissing them may have a positive effect on community-based HIV care as it may reduce stigma and increase cooperation (30, 42). Better referral systems and culturally sensitive counseling may increase adherence with ART regimens (50). Such approaches are essential not only for achieving SDG Target 3.3 but also for addressing the broader health needs of people living with HIV, including the prevention of HIV-related comorbidities and long-term complications (19). A balanced approach is therefore required, especially one that acknowledges the challenges posed by certain sociocultural beliefs while also recognizing documented successes of collaborative engagement with traditional and religious leaders in strengthening HIV outcomes and advancing progress toward SDG Target 3.3.

### Limitations and strengths of the study

This review reports insight on improving HIV health care in different parts of Africa as it ties into the SDG goal Target 3.3. But there are limitations that should be taken into consideration. For one, there was no quality checks on the studies included. The included studies used different methods and were conducted in different locations, thus making it difficult to directly compare the findings. Inclusion of only peer-reviewed studies came with a potential of missing findings from non-peer reviewed materials such as dissertations. Despite these limitations, the strength of this review lies in its integration of diverse evidence sources with policy and health-system perspectives, offering culturally grounded and collaborative insights relevant to achieving SDG Target 3.3 in African settings.

## Conclusion

This review pulls together evidence about how factors such as stigma and religious beliefs, along with mixing different ways of seeking health care amongst PLHIV in some parts of Africa. These factors overlap and affect health-seeking behavior such as testing to starting treatment on time and keeping a low viral load. Thus, making it harder to achieve the SDG for ending epidemics like AIDS by 2030. Stigma, cultural silence, religious and traditional belief systems were found to form part of the broader social structures that shape how PLHIV interpret illness and seek care. As a result, individuals are often diagnosed at a late stage, experience treatment interruptions and disengage from care. Addressing these issues in isolation overlooks their interconnectedness, which collectively impedes progress. Research indicates that traditional healers and religious groups do not always oppose biomedical HIV treatments. This offers an opportunities for collaboration, such as facilitating dialogue between healers and clinics, establishing referral pathways, and partnering with church leaders to reduce stigma and support sustained ART adherence. This approach may offer practical strategies to improve HIV care outcomes without imposing a singular model in African health systems. In addition, formal partnerships and cross-sector training should be provided to align traditional practices with public health objectives. Furthermore, policy should integrate cultural and religious stakeholders into HIV strategies.

## References

[1] Assembly UG. Political declaration on HIV and AIDS: ending inequalities and getting on track to end AIDS by 2030. In: 74th Plenary Meeting. (2021). Available online at: https://www.unaids.org/sites/default/files/media_asset/2021_political-declaration-on-hiv-and-aids_en.pdf (Accessed August 8, 2025).

[2] Micah AE Su Y Bachmeier SD Chapin A Cogswell IE Crosby SW Health sector spending and spending on HIV/AIDS, tuberculosis, and malaria, and development assistance for health: progress towards sustainable development goal 3. Lancet. (2020) 396(10252):693–724. 10.1016/S0140-6736(20)30608-5 32334655 PMC7180045

[3] Abubakar A Van Baar A Fischer R Bomu G Gona JK Newton CR. Socio-cultural determinants of health-seeking behaviour on the Kenyan coast: a qualitative study. PLoS One. (2013) 8(11):e71998. 10.1371/journal.pone.0071998 24260094 PMC3832523

[4] Baxter S Barnes A Lee C Mead R Clowes M. Increasing public participation and influence in local decision-making to address social determinants of health: a systematic review examining initiatives and theories. Local Gov Stud. (2023) 49(5):861–87. 10.1080/03003930.2022.2081551

[5] Martins DC Babajide O Maani N Abdalla SM Gómez EJ Pongsiri MJ Integrating social determinants in decision-making processes for health: insights from conceptual frameworks—the 3-D commission. J Urban Health. (2021) 98(S1):51–9. 10.1007/s11524-021-00560-z 34480328 PMC8415431

[6] Biermann O Mwoka M Ettman CK Abdalla SM Shawky S Ambuko J Data, social determinants, and better decision-making for health: the 3-D commission. J Urban Health. (2021) 98(S1):4–14. 10.1007/s11524-021-00556-9 34414512 PMC8376119

[7] Obeagu EI. Empowering progress: impactful innovations in HIV prevention in Africa. Elite J Public Health. (2024) 2(3):63–77.

[8] Rosenberg NE Gichane MW Vansia D Phanga T Bhushan NL Bekker LG Assessing the impact of a small-group behavioral intervention on sexual behaviors among adolescent girls and young women in Lilongwe Malawi: a quasi-experimental cohort study. AIDS Behav. (2020) 24(5):1542–50. 10.1007/s10461-019-02669-4 31512067 PMC7064392

[9] Brainard D Cihlar T Geleziunas R SenGupta D. HIV: Progress and future challenges in treatment, prevention and cure. Nature. (2018). Available online at: https://www.nature.com/articles/d42473-018-00280-0 (Accessed August 8, 2025).

[10] Onuoha OO Okereke OJ Ngwoke GO Ekpechu JOA. Influence of cultural factors on health seeking behavior in ebonyi state, south east, Nigeria. Niger J Soc Psychol. (2024) 7(2). Available online at: https://www.nigerianjsp.com/index.php/NJSP/article/view/154 (Accessed January 28, 2026).

[11] Ogu RN Agholor KN Okonofua FE. Engendering the attainment of the SDG-3 in Africa: overcoming the socio cultural factors contributing to maternal mortality. Afr J Reprod Health. (2016) 20(3):62–74. 10.29063/ajrh2016/v20i3.11 29553196

[12] Obeagu EI. Breaking barriers: mitigating stigma to control HIV transmission. Elite J Public Health. (2024) 2(8):44–55.

[13] Chambers LA Rueda S Baker DN Wilson MG Deutsch R Raeifar E Stigma, HIV and health: a qualitative synthesis. BMC Public Health. (2015) 15(1):848. 10.1186/s12889-015-2197-0 26334626 PMC4557823

[14] Turan B Hatcher AM Weiser SD MO J Rice WS Turan JM. Framing mechanisms linking HIV-related stigma, adherence to treatment, and health outcomes. Am J Public Health. (2017) 107(6):863–9. 10.2105/AJPH.2017.303744 28426316 PMC5425866

[15] Msoka EF Dwarampudi S Billings R Stone RJ Mwageni RE Beavers A The role of traditional healers along the cancer care continuum in sub-Saharan Africa: a scoping review. Arch Public Health. (2025) 83(1):35. 10.1186/s13690-025-01521-7 39948678 PMC11823172

[16] Patil AD Singh S Verma D Goupale C. Exploring medical pluralism as a multifaceted approach to healthcare. Indian J Integr Med. (2024) 4(2):49–59.

[17] Stoner BP. Understanding medical systems: traditional, modern, and syncretic health care alternatives in medically pluralistic societies. Med Anthropol Q. (1986) 17(2):44–8. 10.1111/j.1937-6219.1986.tb01021.x

[18] Kavinya T. Opinions on the church’s stand against condom use by the youth. Malawi Med J J Med Assoc Malawi. (2009) 21(1):33. PMC3345726 19780477

[19] Bune GT. The impact of sociocultural contexts on the knowledge, attitudes, and practices of adults living with HIV/AIDS in Ethiopia towards metabolic syndrome risks: a descriptive phenomenology study using the PEN-3 model. PLoS One. (2024) 19(8):e0308891. 10.1371/journal.pone.0308891 39172933 PMC11340946

[20] Engler K Lènàrt A Lessard D Toupin I Lebouché B. Barriers to antiretroviral therapy adherence in developed countries: a qualitative synthesis to develop a conceptual framework for a new patient-reported outcome measure. AIDS Care. (2018) 30(sup1):17–28. 10.1080/09540121.2018.1469725 29719990

[21] Misir P. Structuration theory: a conceptual framework for HIV/AIDS stigma. J Int Assoc Provid AIDS Care JIAPAC. (2015) 14(4):328–34. 10.1177/2325957412463072 26195671

[22] Ijiga OM Ifenatuora GP Olateju M. Bridging STEM and cross-cultural education: designing inclusive pedagogies for multilingual classrooms in sub Saharan Africa. IRE J. (2021) 5(1):237–52.

[23] Olana DE. Socio-cultural perspectives of HIV/AIDS communication amongst the Borana pastoralist community in Ethiopia. Phd thesis. South Africa, University of South Africa; (2024). Available online at: https://search.proquest.com/openview/f5ac71c877ede83c83dddce1af7e2b79/1?pq-origsite=gscholar&cbl=2026366&diss=y (Accessed January 28, 2026).

[24] Williams LD. Understanding the relationships among HIV/AIDS-related stigma, health service utilization, and HIV prevalence and incidence in sub-Saharan Africa: a multi-level theoretical perspective. Am J Community Psychol. (2014) 53(1–2):146–58. 10.1007/s10464-014-9628-4 24477769

[25] Chipfakacha VG. STD/HIV/AIDS knowledge, beliefs and practices of traditional healers in Botswana. AIDS Care. (1997) 9(4):417–25. 10.1080/713613174 9337886

[26] Menze N Mashigo T. Role of traditional healers in healthcare in the 21st century. Curr Allergy Clin Immunol. (2025) 38(4):2–4.

[27] Moshabela M Bukenya D Darong G Wamoyi J McLean E Skovdal M Traditional healers, faith healers and medical practitioners: the contribution of medical pluralism to bottlenecks along the cascade of care for HIV/AIDS in eastern and Southern Africa. Sex Transm Infect. (2017) 93(Suppl 3):e052974. 10.1136/sextrans-2016-052974 28736393 PMC5739844

[28] Galvin M Chiwaye L Moolla A. Religious and medical pluralism among traditional healers in Johannesburg, South Africa. J Relig Health. (2024) 63(2):907–23. 10.1007/s10943-023-01795-7 36971902 PMC10040931

[29] Ozioma EOJ Chinwe OAN. Herbal medicines in African traditional medicine. In: Herbal Medicine. London: IntechOpen (2019). Available online at: https://books.google.com/books?hl=en&lr=&id=AGmQDwAAQBAJ&oi=fnd&pg=PA191&dq=Traditional+healers+remain+the+first+contact+for+most+African+individuals&ots=mXOLnpWcDW&sig=QV7F400xpStJmvsYGPO07T0KggU (Accessed September 20, 2025).

[30] Mutambara J Sodi T Mtemeri J Makomo M. Harmonizing religion and health: an exploration of religious reasons for defaulting ARVs among people living with HIV and AIDS in Gweru, Zimbabwe. AIDS Care. (2021) 33(3):383–8. 10.1080/09540121.2020.1724255 32030992

[31] Schwarcz S Richards T Anne F Heidi W Conrad C Hsu L Identifying barriers to HIV testing: personal and contextual factors associated with late HIV testing. AIDS Care. (2011) 23(7):892–900. 10.1080/09540121.2010.534436 21424942

[32] Roura M Nsigaye R Nhandi B Wamoyi J Busza J Urassa M “Driving the devil away”: qualitative insights into miraculous cures for AIDS in a rural Tanzanian ward. BMC Public Health. (2010) 10(1):427. 10.1186/1471-2458-10-427 20646300 PMC2916904

[33] Pitman MC Lau JS McMahon JH Lewin SR. Barriers and strategies to achieve a cure for HIV. Lancet HIV. (2018) 5(6):e317–28. 10.1016/S2352-3018(18)30039-0 29893245 PMC6559798

[34] Jabr AM Stefano MD Greco P Santantonio T Fiore JR. Errors in condom use in the setting of HIV transmission: a systematic review. Open AIDS J. (2020) 14(1):16–26. 10.2174/1874613602014010016

[35] Wambua GN. Knowledge, attitude and practices on pre-exposure prophylaxis among HIV infected and aids discordant couples in Kitui west sub-county, Kitui county, Kenya. Phd thesis. (2024). Available online at: https://repository.seku.ac.ke/handle/123456789/7709 (Accessed September 20, 2025).

[36] Organization WH. Ending the Neglect to Attain the Sustainable Development Goals: A Rationale for Continued Investment in Tackling Neglected Tropical Diseases 2021–2030. Geneva: World Health Organization (2022). Available online at: https://books.google.com/books?hl=en&lr=&id=Vw2MEAAAQBAJ&oi=fnd&pg=PR9&dq=Therefore,+to+end+the+epidemic+of+AIDS,+tuberculosis,+malaria,+and+neglected+tropical+diseases+by+2030+(Sustainable+Development+Goal+Target+3.3),+health-seeking+behavior+specific+to+the+communities+should+be+considered+when+engaging+the+community+for+better+outcomes+of+HIV+management&ots=UiTpuHTiH_&sig=M89G50Gm0ZRS1es-MmALhrE46EQ (Accessed September 20, 2025).

[37] Syed IA Sulaiman SAS Hassali MA Thiruchelvam K Syed SH Lee CK. Beliefs and practices of complementary and alternative medicine (CAM) among HIV/AIDS patients: a qualitative exploration. Eur J Integr Med. (2016) 8(1):41–7. 10.1016/j.eujim.2015.09.135

[38] Bene M Darkoh MBK. The constraints of antiretroviral uptake in rural areas: the case of Thamaga and surrounding villages, Botswana. SAHARA J J Soc Asp HIVAIDS Res Alliance. (2014) 11(1):167–77. 10.1080/17290376.2014.972057 PMC4272138 25365702

[39] Shuster JM Sterk CE Frew PM del Rio C. The cultural and community-level acceptance of antiretroviral therapy (ART) among traditional healers in eastern cape, South Africa. J Community Health. (2009) 34(1):16–22. 10.1007/s10900-008-9121-9 18923887

[40] Beressa TB Deyno S Mtewa AG Aidah N Tuyiringire N Lukubye B Potential benefits of antiviral African medicinal plants in the management of viral infections: systematic review. Front Pharmacol. (2021) 12:682794. 10.3389/fphar.2021.682794 35002686 PMC8740180

[41] Salehi B Kumar NVA Şener B Sharifi-Rad M Kılıç M Mahady GB Medicinal plants used in the treatment of human immunodeficiency virus. Int J Mol Sci. (2018) 19(5):1459. 10.3390/ijms19051459 29757986 PMC5983620

[42] Zou J Yamanaka Y John M Watt M Ostermann J Thielman N. Religion and HIV in Tanzania: influence of religious beliefs on HIV stigma, disclosure, and treatment attitudes. BMC Public Health. (2009) 9:75. 10.1186/1471-2458-9-75 19261186 PMC2656538

[43] Raja M Cramer H Lee MS Wieland LS Ng JY. Addressing the challenges of traditional, complementary, and integrative medicine research: an international perspective and proposed strategies moving forward. Perspect Integr Med. (2024) 3(2):86–97. 10.56986/pim.2024.06.004

[44] von Schoen-Angerer T Manchanda RK Lloyd I Wardle J Szöke J Benevides I Traditional, complementary and integrative healthcare: global stakeholder perspective on WHO’s current and future strategy. BMJ Glob Health. (2023) 8(12). 10.1136/bmjgh-2023-013150 38050407 PMC10693890

[45] Liang Z Lai Y Li M Shi J Lei CI Hu H Applying regulatory science in traditional Chinese medicines for improving public safety and facilitating innovation in China: a scoping review and regulatory implications. Chin Med. (2021) 16(1):23. 10.1186/s13020-021-00433-2 33593397 PMC7884970

[46] Ijaz N Boon H. Statutory regulation of traditional medicine practitioners and practices: the need for distinct policy making guidelines. J Altern Complement Med. (2018) 24(4):307–13. 10.1089/acm.2017.0346 29359948 PMC5909079

[47] Runnacles J Roueché A Lachman P. The right care, every time: improving adherence to evidence-based guidelines. Arch Dis Child Educ Pract. (2018) 103(1):27–33. 10.1136/archdischild-2017-312740 28536137

[48] Moshabela M Zuma T Gaede B. Bridging the gap between biomedical and traditional health practitioners in South Africa. South Afr Health Rev. (2016) 2016(1):83–92.

[49] Nyembezi A Ngcobo S Lehmann U. Collaboration between traditional health practitioners and biomedical health practitioners: scoping review. Afr J Prim Health Care Fam Med. (2024) 16(1):1–11. 10.4102/phcfm.v16i1.4430 PMC11304181 39099280

[50] Audet CM Clemens EM Ngobeni S Mkansi M Sack DE Wagner RG. Throwing the bones to diagnose HIV: views of rural South African traditional healers on undertaking HIV counselling and testing. AIDS Care. (2021) 33(10):1316–20. 10.1080/09540121.2020.1808568 32799661 PMC7887123

[51] Wits University. 2023-08—Traditional healers in rural Mpumalanga help diagnose HIV—Wits University. Available online at: https://www.wits.ac.za/news/latest-news/research-news/2023/2023-08/traditional-healers-in-rural-mpumalanga-help-diagnose-hiv.html (Accessed June 6, 2025).

[52] Peltzer K Preez NF du Ramlagan S Fomundam H. Use of traditional complementary and alternative medicine for HIV patients in KwaZulu-Natal, South Africa. BMC Public Health. (2008) 8(1):255. 10.1186/1471-2458-8-255 18652666 PMC2503977

[53] Khangale N. Traditional healers: trained to identify signs of HIV/AIDS|Vuk’uzenzele. Available online at: https://www.vukuzenzele.gov.za/traditional-healers-trained-identify-signs-hivaids (Accessed June 6, 2025).

[54] Matege A. Traditional Healers Join Uganda’s HIV Fight in Groundbreaking Health Project|The Kampala Post (2025). Available online at: https://kampalapost.com/content/traditional-healers-join-ugandas-hiv-fight-groundbreaking-health-project (Accessed June 6, 2025).

[55] Mabunda A Madiba S. The context of parent-child communication about sexuality and HIV prevention; the perspectives of high school learners in gauteng province, South Africa. (2017). Available online at: https://ubrisa.ub.bw/handle/10311/2116 (Accessed September 20, 2025).

[56] Amissah-Arthur MB Gyaban-Mensah A Boima V Yorke E Dey D Ganu V Health-seeking behaviour, referral patterns and associated factors among patients with autoimmune rheumatic diseases in Ghana: a cross-sectional mixed method study. PLoS One. (2022) 17(9):e0271892. 10.1371/journal.pone.0271892 36094929 PMC9467363

[57] Msoka EF Abraham M Mulder BC Beavers A Gebremariam A Bright FB The perspectives of healthcare providers, traditional healers, and other key informants on the late diagnosis of breast cancer in northern Tanzania: a qualitative study. Discov Public Health. (2025) 22(1):527. 10.1186/s12982-025-00920-z

[58] Obeagu EI Obeagu GU. Moving forward together: collaborative strategies in HIV prevention across Africa–a narrative review. Ann Med Surg. (2025) 87(7):4117–26. 10.1097/MS9.0000000000002961 PMC12369799 40852004

[59] Low JJQ Ayoko OB. The emergence of spiritual leader and leadership in religion-based organizations. J Bus Ethics. (2020) 161(3):513–30. 10.1007/s10551-018-3954-7

[60] Moshabela M Bukenya D Darong G Wamoyi J McLean E Skovdal M Traditional healers, faith healers and medical practitioners: the contribution of medical pluralism to bottlenecks along the cascade of care for HIV/AIDS in eastern and Southern Africa. Sex Transm Infect. (2017) 93(Suppl 3). 10.1136/sextrans-2016-052974 PMC5739844 28736393

[61] Mioro JA. Stigma and discrimination against people living with hiv (plhiv) in the methodists church a case of njia circuit, igembe south sub county, meru county. Phd thesis. Kenyatta University; (2015). Available online at: https://ir-library.ku.ac.ke/bitstreams/044f5244-ae63-4868-bb14-faa97f7e2715/download (Accessed September 20, 2025).

[62] Gaia JB. Moral issues and responsibilities regarding HIV/AIDS. Mission South Afr J Mission Stud. (2002) 30(2):265–87.

[63] Wolf HT Halpern-Felsher BL Bukusi EA Agot KE Cohen CR Auerswald CL. It is all about the fear of being discriminated [against]…the person suffering from HIV will not be accepted”: a qualitative study exploring the reasons for loss to follow-up among HIV-positive youth in Kisumu, Kenya. BMC Public Health. (2014) 14(1):1154. 10.1186/1471-2458-14-1154 25377362 PMC4232620

[64] Mosley EA Narasimhan S Blevins J Dozier JL Pringle J Clarke LS Sexuality-based stigma and inclusion among southern protestant religious leaders. Sex Res Soc Policy. (2022) 19(4):1519–32. 10.1007/s13178-021-00662-y

[65] Skinner D Mfecane S. Stigma, discrimination and the implications for people living with HIV/AIDS in South Africa. SAHARA-J J Soc Asp HIVAIDS. (2004) 1(3):157–64. 10.1080/17290376.2004.9724838 17601003

[66] Treves-Kagan S Steward WT Ntswane L Haller R Gilvydis JM Gulati H Why increasing availability of ART is not enough: a rapid, community-based study on how HIV-related stigma impacts engagement to care in rural South Africa. BMC Public Health. (2015) 16(1):87. 10.1186/s12889-016-2753-2 PMC4730651 26823077

[67] Tohit NFM Haque M. Forbidden conversations: a comprehensive exploration of taboos in sexual and reproductive health. Cureus. (2024) 16(8). 10.7759/cureus.66723 PMC11319820 39139803

[68] Patil CL Klima CS Steffen AD Leshabari SC Pauls H Norr KF. Implementation challenges and outcomes of a randomized controlled pilot study of a group prenatal care model in Malawi and Tanzania. Int J Gynaecol Obstet Off Organ Int Fed Gynaecol Obstet. (2017) 139(3):290–6. 10.1002/ijgo.12324 PMC5673548 28905377

[69] Mabunda D Oliveira D Sidat M Cavalcanti MT Cumbe V Mandlate F Cultural adaptation of psychological interventions for people with mental disorders delivered by lay health workers in Africa: scoping review and expert consultation. Int J Ment Health Syst. (2022) 16(1):14. 10.1186/s13033-022-00526-x 35168650 PMC8845308

