# Supplementary material for: Navigating sociocultural practices and traditions in HIV management: a review of African cultural barriers to achieving sustainable development goal target 3.3
Source: Front Epidemiol. 2026 Mar 5;6:1710531. doi: 10.3389/fepid.2026.1710531 (PMC12999944; doi:10.3389/fepid.2026.1710531)
Supplement: Supplementary file 1 [file Table1.docx]

**Navigating sociocultural practices and traditions in HIV management: A review of African cultural barriers to achieving** **Sustainable Development Goal Target 3.3.**

Reneilwe G Mashaba^1^*, Cairo B Ntimana ^1,2*^

^1^ DIMAMO Population Health Research Centre, University of Limpopo, Sovenga St, Polokwane 0727, South Africa

2 Department of Pathology, University of Limpopo, Sovenga St, Polokwane 0727, South Africa

* Correspondence: [given.mashaba@ul.ac.za](mailto:given.mashaba@ul.ac.za); [cairo.ntimane@ul.ac.za](mailto:cairo.ntimane@ul.ac.za)

Table S1: study characteristics

| **Authors (year)** | **Location** | **Methodological approach** | **Key findings** |
| --- | --- | --- | --- |
| [Chipfakacha](https://www.tandfonline.com/author/CHIPFAKACHA%2C+V+G) et al., 1997(1) | Botswana | Cross-sectional | Most of the patients who are discharged from hospitals on home-based care usually end up at the traditional healer as relatives seek a second opinion or simply because they disagree with the diagnosis of incurable disease. |
| Moshabela et al., 2016(2) | South Africa | Cross-sectional | Medical pluralism, manifesting across traditional, faith-based and biomedical health-worlds, contributed to the care cascade bottlenecks for PLHIV through three pathways of impact. First, access to HIV treatment was delayed through the nature of health-related beliefs, knowledge and patient journeys. Second, HIV treatment was interrupted by availability of alternative options, perceived failed treatment and exploitation of PLHIV by opportunistic traders and healers. Lastly, the mixing of biomedical healthcare providers and treatment with traditional and faith-based options fuelled tensions driven by fear of drug-to-drug interactions and mistrust between providers operating in different health-worlds. |
| Galvin et al., 2024(3) | South Africa | Qualitative | Many THPs also often practice medical pluralism, mixing Western treatments with traditional practices/medicines |
| Mutambara et al., 2019(4) | Zimbabwe | Qualitative | PLWHIV defaulted medications because – they believed in faith healing, alternative medicines, perceived spirituality as the main cause of HIV and AIDS and that they had an allegiance to church values. |
| Roura et al., 2010(5) | Tanzania | Qualitative | Traditional healers and FLs were often reported as antagonists but duality prevailed and many FLs simultaneously believed in traditional healing. Inter-denomination mobility was high and guided by pragmatism. Praying for the sick was a common practice and over one third of respondents said that prayer could cure HIV. Being HIV-positive was often seen as "a punishment from God" and a consequence of sin. As sinning could result from "the work of Satan", forgiveness was possible, and a "reconciliation with God" deemed as essential for a favourable remission of the disease. Several FLs believed that "evil spirits" inflicted through witchcraft could cause the disease and claimed that they could cast "demons" away. While prayers could potentially cure HIV "completely", ART use was generally not discouraged because God had "only a part to play". The perceived potential superiority of spiritual options could however lead some users to interrupt treatment. |
| Bene et al., 2014(6) | Botswana | Cross-sectional | Some of the people living with HIV believed that ARV therapy could better their lives during the initial stages of introduction, but with time, they lost hope and gave up the treatment. Culturally, parents and children in the villages do not discuss sexual matters at home and it was found in the study that there was little communication between parents and children on AIDS and ARV issues. Some churches in the area discouraged the use of ARV. There were also traditional doctors who made their patients mix traditional herbs treatment with ARV treatment. Distance, travel costs, cultural beliefs, stigma and discrimination among others were found to be important socio-economic factors inhibiting ARV uptake. |
| Shuster et al., 2008(7) | South Africa | Qualitative | By obtaining the opinions of traditional healers currently interested in biomedical approaches to HIV/AIDS care and prevention, this formative investigation identified a range of motivational factors that were believed to promote a deeper acceptance of and support for ART. These factors included cultural consistencies between traditional and biomedical medicine, education, as well as legal and financial incentives to collaborate |
| Zou et al., 2009(8) | Tanzania | Cross-sectional | Results indicate that shame-related HIV stigma is strongly associated with religious beliefs such as the belief that HIV is a punishment from God or that people living with HIV/AIDS (PLWHA) have not followed the Word of God. Most participants (84.2%) said that they would disclose their HIV status to their pastor or congregation if they became infected. Although most respondents (80.8%) believed that prayer could cure HIV, almost all (93.7%) said that they would begin ARV treatment if they became HIV-infected. The multivariate analysis found that respondents' hypothetical willingness to begin ARV treatment was not significantly associated with the belief that prayer could cure HIV or with other religious factors. Refusal of ARV treatment was instead correlated with lack of secondary schooling and lack of knowledge about ARV. |
| Audet et al., 2020(9) | South | Qualitative |  |
| Peltzer et al., 2008(10) | South Africa | Cross-sectional | Herbal therapies were the most expensive, costing on average 128 Rand per patient per month. Most participants indicated that their health care provider was not aware that they were taking herbal therapies for HIV (90%). Herbal therapies were mainly used for pain relief (87.1%) and spiritual practices or prayer for stress relief (77.6%). Multivariate logistic regression with use of herbs for HIV as the dependent variable identified being on a disability grant and fewer clinic visits to be associated with use of herbs, and TCAM use for HIV identified being on a disability grant, number of HIV symptoms and family members not contributing to main source of household income to be associated with TCAM use. |
| Mabunda et al., 2017(11) | South Africa | Qualitative | The findings revealed that communication between parents and adolescents was not planned, was infrequent, and characterized by warnings and threats. The tone of the communication was harsh, ambiguous, and filled with threats and warnings about the dangers of HIV. The adolescents felt that the communication was unidirectional, took the form of a lecture rather than dialogue; the information was at times incorrect, and the consequences of sex were exaggerated. They perceived the tone of the discussions as a barrier to effective communication with their parents. The discussions were triggered by various factors like the perceived undesirable behavior by the adolescents, the parental suspicion of sexual debut, puberty, and menstrual flow. |
| Msoka et al., 2025(12) | Tanzania | Qualitative | The findings were synthesized into five themes to explain late diagnosis: (1) HCPs and THs have limited knowledge and misconceptions about the causes, signs, and symptoms of breast cancer; (2) patients experience stigma (3) treatment is expensive and difficult to access; (4) patients perceive barriers based on myths, misperceptions and spiritual beliefs; and (5) HCPs and THs often blame each other for ineffective treatment and delays in effective treatment. The participants also provided suggestions to reduce delays in diagnosis and treatment, including enhancing knowledge and awareness among THs and HCPs to help them recognize the signs and symptoms of BC through education campaigns, addressing the cost of access to care and treatment, addressing stigma associated with BC, and developing collaborative efforts between HCPs and THs. |
| Mosa et al., 2017 (13) |  |  | Medical pluralism, manifesting across traditional, faith-based and biomedical health-worlds, contributed to the care cascade bottlenecks for PLHIV through three pathways of impact. First, access to HIV treatment was delayed through the nature of health-related beliefs, knowledge and patient journeys. Second, HIV treatment was interrupted by availability of alternative options, perceived failed treatment and exploitation of PLHIV by opportunistic traders and healers. Lastly, the mixing of biomedical healthcare providers and treatment with traditional and faith-based options fuelled tensions driven by fear of drug-to-drug interactions and mistrust between providers operating in different health-worlds. |
| Wolf et al., 2014(14) | Kenya | Qualitative | HIV-related stigma was the overarching factor that led to LTFU among HIV + youth. Stigma operated on multiple levels to influence LTFU, including in the home/family, at school, and at the clinic. In all three settings, participants’ fear of stigma due to disclosure of their HIV status contributed to LTFU. Likewise, in the three settings, the dependent relationships between youth and the key adult figures in their lives were also adversely impacted by stigma and resultant lack of disclosure. Thus, at all three-settings stigma influenced fear of disclosure, which in turn impacted negatively on dependent relationships with adults on whom they rely (i.e. parents, teachers and clinicians) leading to LTFU. |
| Audet et al., 2020(15) | South Africa | Qualitative | Healers reported that while some patients are open about their HIV status, others lie about it due to stigma. This creates challenges with concurrent treatment, which healers believe leads to allopathic and/or traditional medication treatment failure. Most healers expressed both an interest and a willingness to perform HIV counseling and testing. Healers felt that by performing testing in the community, it would overcome issues related to HIV stigma, as well as a lack of confidentiality and trust with health care workers at the clinic. Trained traditional healers may be able to bridge the testing gap between “non-testers” and the allopathic health system, essentially “opening” thousands of new testing locations with little financial investment. |
| Skinners et al., (16) | South Africa | Qualitative | Stigma also introduces a desire not to know one's own status, thus delaying testing and accessing treatment. At an individual level stigma undermines the person's identity and capacity to cope with the disease. Fear of discrimination limits the possibility of disclosure even to potential important sources of support such as family and friends. Finally, stigma impacts on behaviour change as it limits the possibility of using certain safer sexual practices. Behaviour such as wanting to use condoms could be seen as a marker of HIV, leading to rejection and stigma. |
| Treves-Kagan et al., 2015(17) | South Africa | Qualitative | Findings suggested that anticipated stigma remains a barrier to care. Although participants reported less enacted stigma, or hostility toward people living with HIV, they also felt that HIV remains synonymous with promiscuity and infidelity. Participants described community members taking steps to avoid being identified as HIV-positive, including avoiding healthcare facilities entirely, using traditional healers, or paying for private doctors. Such behaviors led to delays in testing and accessing care, and problems adhering to medications, especially for men and youth with no other health condition that could plausibly account for their utilization of medical services. |
